# From Epigenetic Constraint to Evolutionary Escape: Cell-State Transitions and Selective Pressures During Malignant Transformation in Lower-Grade Gliomas

**DOI:** 10.3390/biomedicines14050985

**Published:** 2026-04-25

**Authors:** Hao Wu, Yi Wei, Xing-Ding Zhang, Lin Qi

**Affiliations:** 1 Guangxi Key Laboratory of Tumor Immunology and Microenvironmental Regulation, Guilin Medical University, Guilin 541199, China; wuhao237@mail2.sysu.edu.cn (H.W.); 18999754630@163.com (Y.W.); 2 Shenzhen Key Laboratory for Systems Medicine in Inflammatory Diseases, School of Medicine, Sun Yat-Sen University, Shenzhen 518106, China; zhangxd39@mail.sysu.edu.cn; 3 Guangxi Health Commission Key Laboratory of Tumor Immunology and Receptor-Targeted Drug Basic Research, Guilin Medical University, Guilin 541199, China

**Keywords:** lower-grade glioma, malignant transformation, cell-state transitions, epigenetic constraint, tumor evolution, selective pressures, tumor microenvironment, IDH-mutant glioma

## Abstract

Lower-grade gliomas (LGGs) often follow a relatively protracted clinical course; however, a substantial proportion eventually undergo malignant transformation to high-grade, treatment-refractory disease. This process has traditionally been interpreted in the context of stepwise histopathologic progression and recurrent genetic alterations. Increasing evidence, however, suggests that malignant transformation is more accurately understood as an evolutionary process shaped by the interplay among epigenetic constraints, cell-state plasticity, and selective pressures. In this review, we examine current evidence supporting a model in which early LGGs, particularly isocitrate dehydrogenase (IDH)-mutant tumors, are initially maintained in relatively restricted cellular states by metabolically imposed epigenetic programs, but progressively escape these constraints under the cumulative influence of therapy, hypoxia, immune remodeling, and genomic instability. We summarize recent advances demonstrating that progression from lower-grade to high-grade disease is accompanied by cell-state transitions characterized by altered lineage identity, acquisition of stem-like features, increased proliferative capacity, and adaptation to cellular stress. We further discuss how these transitions are reinforced by microenvironmental evolution, including vascular remodeling, extracellular matrix reorganization, and changes in immune composition, thereby creating conditions that favor clonal expansion, invasion, and therapeutic resistance. Particular attention is given to longitudinal, single-cell, and spatially resolved studies, which collectively indicate that malignant transformation is not a discrete event but a continuous process of evolutionary selection and phenotypic reprogramming. Finally, we discuss the translational implications of this framework for early risk stratification, biomarker development, and mechanism-based therapeutic intervention. By reframing malignant transformation in LGGs as a process of cell-state escape under persistent selective pressure, this review aims to provide an integrated view of glioma progression and to highlight new opportunities for precision monitoring and treatment.

## 1. Introduction

Lower-grade gliomas (LGGs) comprise a biologically heterogeneous group of diffuse gliomas characterized by variable clinical trajectories and a persistent risk of malignant progression. Within the current WHO framework, adult diffuse gliomas are defined by integrated histologic and molecular criteria, most notably isocitrate dehydrogenase (*IDH*) mutation status and *1p/19q* codeletion. Although this classification has substantially improved disease stratification, it has not resolved a central question in neuro-oncology: why do some LGGs remain relatively indolent for prolonged periods, whereas others undergo malignant transformation into highly proliferative, invasive, and treatment-refractory disease? Existing clinical, radiographic, and molecular predictors provide useful associations, but they do not, by themselves, offer a coherent mechanistic account of how transformation unfolds over time. Rather than a discrete event, malignant transformation is increasingly recognized as a temporally extended process shaped by the interaction of tumor-intrinsic programs, cell-state plasticity, and microenvironmental selection [1].

### 1.1. Epidemiology and Clinical Burden of LGGs

Gliomas account for approximately 30% of all primary brain tumors and nearly 80% of malignant brain tumors. Within this spectrum, lower-grade diffuse gliomas are less common than glioblastoma but remain clinically important because they typically affect younger adults, often follow a prolonged course, and frequently recur with progressive biological evolution. Recent population-based analyses estimate the age-adjusted incidence of *IDH*-mutant glioma at approximately 0.8 per 100,000 persons in the United States, underscoring that these tumors, although less frequent than glioblastoma, represent a substantial long-term source of morbidity and mortality in adult neuro-oncology [2,3].

### 1.2. Risk Factors Associated with Glioma Development

Established risk factors for glioma remain limited. Exposure to ionizing radiation is the most consistently validated environmental risk factor, whereas inherited cancer-predisposition syndromes account for only a minority of cases. Broader epidemiologic studies have also identified associations with age, sex, and possible inverse relationships with atopy or allergic disease, although risk factors specific to adult-type lower-grade gliomas remain incompletely defined. This limited etiologic understanding further emphasizes the importance of clarifying the biological mechanisms that govern progression and malignant transformation once LGGs have arisen [2,4].

### 1.3. How LGGs Differ from Other Glioma Subtypes

Adult-type lower-grade gliomas, especially *IDH*-mutant astrocytomas and oligodendrogliomas, differ fundamentally from *IDH*-wild-type glioblastoma in age distribution, molecular architecture, natural history, and treatment response. They are enriched in younger adults, are shaped by mutant *IDH*-associated metabolic and epigenetic reprogramming, and typically show a more protracted course before recurrence or grade progression. These distinctions are important because mechanisms derived from glioblastoma cannot be assumed to apply directly to LGGs, particularly when discussing transformation, cell-state plasticity, and microenvironmental adaptation [2,5].

### 1.4. Malignant Transformation as an Evolving Process

Historically, malignant transformation in LGGs has been interpreted through two dominant lenses. The first is a histopathologic model, in which progression is viewed as a stepwise increase in grade defined by mitotic activity, microvascular proliferation, necrosis, and other morphologic hallmarks. The second is a genetic progression model, in which recurrent disease is understood primarily as the consequence of additional driver alterations superimposed on an initiating glioma genotype. Both perspectives have been indispensable, but each is incomplete when considered in isolation. Histology captures biologic endpoints rather than continuous evolutionary change, while mutation-centered models do not fully explain how tumors with similar genetic backbones can display markedly different trajectories of progression, latency, and treatment response [1,6].

Recent longitudinal, single-cell, and spatially resolved studies have begun to reshape this view. Instead of representing a discrete event, malignant transformation increasingly appears to reflect a dynamic process in which tumor cell identity, developmental state, and ecological fitness are progressively remodeled under therapeutic and microenvironmental pressure. Paired primary–recurrent analyses have shown that glioma progression is shaped not only by genetic evolution but also by reciprocal interactions with the tumor microenvironment [6]. More recently, high-resolution profiling of *IDH*-mutant gliomas has revealed reproducible shifts in malignant cell populations during progression, including transitions from relatively restricted, slow-cycling states toward more proliferative and developmentally plastic states [7]. These findings suggest that transformation is not simply the late appearance of more aggressive clones, but the cumulative outcome of selective forces acting on cellular states that are initially constrained and later progressively destabilized.

### 1.5. Conceptual Framework of This Review

**Figure 1:**
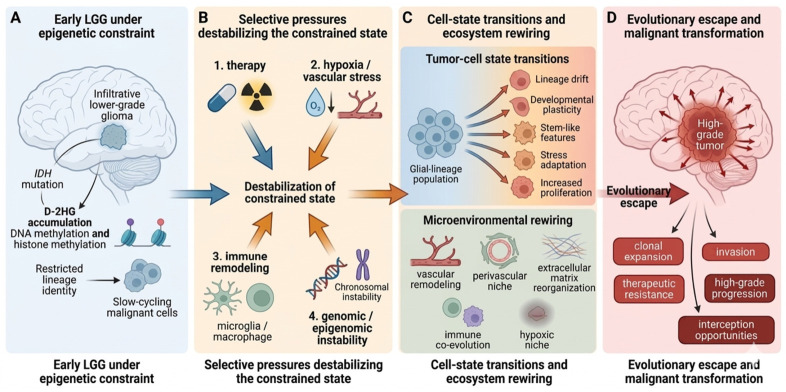
From epigenetic constraint to evolutionary escape: a conceptual framework for malignant transformation in lower-grade gliomas. Early lower-grade gliomas (LGGs), particularly *IDH*-mutant tumors, may initially occupy relatively restricted cellular states maintained by metabolically driven epigenetic programs (**A**). Over time, persistent selective pressures-including therapy, hypoxia/vascular stress, immune remodeling, and genomic or epigenomic instability-progressively destabilize this constrained state (**B**). Under these conditions, tumor cells undergo cell-state transitions marked by lineage drift, increased developmental plasticity, acquisition of stem-like features, stress adaptation, and enhanced proliferative capacity, while the surrounding ecosystem is concurrently rewired through vascular remodeling, perivascular niche formation, extracellular matrix reorganization, hypoxic adaptation, and reciprocal evolution with the immune microenvironment (**C**). Malignant transformation emerges as the cumulative outcome of these processes, resulting in clonal expansion, invasion, therapeutic resistance, and progression to high-grade disease (**D**).

In this Review, we propose a framework in which malignant transformation in LGGs is conceptualized as a transition from epigenetic constraint to evolutionary escape. In early-stage LGGs, particularly IDH-mutant tumors, metabolically imposed epigenetic programs appear to help maintain malignant cells within comparatively restricted lineage and proliferative states. Over time, however, this constrained condition is eroded by persistent selective pressures, including therapy, hypoxia and vascular stress, immune remodeling, and genomic or epigenomic instability. In parallel, ecosystem rewiring through vascular adaptation, extracellular matrix reorganization, and niche remodeling further reinforces aggressive tumor states. Transformation emerges when these pressures enable sustained cell-state transitions toward stem-like, stress-adapted, proliferative, and invasion-permissive phenotypes. By integrating evidence from molecular pathology, tumor ecology, and longitudinal profiling, we argue that this framework offers a more biologically coherent explanation for LGG progression than static grade-based or mutation-only models. It also provides a useful basis for identifying earlier biomarkers of state instability and for designing interventions aimed not only at tumor cytoreduction, but also at preventing or delaying evolutionary escape [1,8,9,10]. Given the current WHO classification and the availability of longitudinal molecular data, this Review focuses primarily on adult-type IDH-mutant lower-grade gliomas, while drawing selected mechanistic insights from the broader diffuse glioma and glioblastoma literature where relevant. The conceptual framework proposed in this Review is summarized in Figure 1.

## 2. Defining the Core Conceptual Terms

In this Review, the terms epigenetic constraint and evolutionary escape are used as integrative conceptual frameworks rather than as single experimentally measurable variables. By epigenetic constraint, we refer to a relatively restricted malignant state in which tumor behavior remains shaped by metabolically imposed epigenetic regulation, preservation of lineage-associated transcriptional programs, and limited developmental plasticity [7,8]. In practical terms, this state may be inferred from features such as methylation-centered regulatory dependence, maintenance of differentiated glial-lineage signatures, relatively low proliferative activity, and enrichment of more restricted malignant cell states in longitudinal or single-cell studies.

By evolutionary escape, we refer to the transition from this relatively restricted state toward a more aggressive and adaptive configuration characterized by loss of prior lineage restriction, expansion of proliferative or progenitor-like populations, increased developmental plasticity, treatment-associated diversification, and progressive remodeling of the tumor ecosystem [7]. Importantly, we do not use this term to imply a single molecular switch or a universally accepted threshold. Rather, it describes a trajectory in which multiple biological features converge to indicate that the tumor is no longer primarily maintained within its earlier constrained state. Similarly, selection in this context refers to the differential fitness of tumor cell states under specific pressures, including therapy, hypoxia, immune remodeling, and ecological change. The term adaptive landscape is used to describe the range of phenotypic states available to the tumor under a given genetic, epigenetic, and microenvironmental context. Neither concept is currently reducible to a single biomarker or numerical cutoff in clinical practice. Instead, they are best approximated through convergent evidence from histopathology, longitudinal molecular profiling, single-cell and spatial analyses, and treatment-associated evolutionary patterns.

Accordingly, the framework proposed here should be viewed as a biologically grounded interpretive model that links measurable observations to a broader evolutionary view of progression, rather than as a rigid classification system defined by fixed thresholds.

## 3. Early LGGs as Epigenetically Constrained Neoplasms

### 3.1. Types of Epigenetic Dysregulation in LGGs

Epigenetic dysregulation in lower-grade gliomas is not limited to a single layer of chromatin control. In IDH-mutant gliomas, the accumulation of D-2-hydroxyglutarate (*D-2HG*) affects multiple α-ketoglutarate-dependent dioxygenases and leads to widespread changes in DNA methylation, histone methylation, and chromatin accessibility. These alterations contribute to the glioma-CpG island methylator phenotype, perturb lineage-associated transcriptional regulation, and may reinforce differentiation blockade and state restriction. Thus, the concept of epigenetic constraint in LGGs should be understood as arising from coordinated abnormalities in DNA methylation, histone-associated regulation, and broader chromatin-state organization rather than from a single epigenetic lesion [8,11].

### 3.2. IDH Mutations and the Metabolic–Epigenetic Architecture of LGGs

The concept of epigenetic constraint provides a useful starting point for understanding why many LGGs initially follow a relatively protracted course. *IDH1* and *IDH2* mutations are among the earliest and most defining events in diffuse gliomagenesis. Their biological consequences extend far beyond classification. Mutant *IDH* enzymes generate the oncometabolite *D-2HG*, which perturbs α-ketoglutarate-dependent dioxygenases involved in DNA demethylation, histone modification, chromatin accessibility, hypoxia signaling, and broader transcriptional regulation. As a result, *IDH*-mutant gliomas develop a distinctive epigenetic landscape that influences differentiation programs, metabolic wiring, and treatment response. This biology helps to explain why *IDH*-mutant LGGs are clinically and molecularly distinct from *IDH*-wild-type high-grade gliomas: they are not simply slower-growing versions of the same disease, but tumors occupying a different regulatory state [7,8].

### 3.3. Epigenetic Constraint and Lineage Restriction

It is therefore insufficient to describe early LGGs only in terms of reduced proliferation or delayed recurrence. A more mechanistic view is that early LGGs are maintained in relatively restricted cellular states by metabolically imposed epigenetic programs. These programs do not make the tumors benign; rather, they impose a constrained biological configuration in which lineage identity, chromatin state, and transcriptional output remain at least partially ordered. This helps account for the paradox that *IDH*-mutant gliomas can persist for years while still retaining malignant potential. The tumor is not dormant, but it is not yet fully released into the broader phenotypic search space that characterizes aggressive recurrent disease. In this sense, the notion of epigenetic constraint is not a rhetorical construct; it reflects the idea that malignant potential is present early, but its expression is initially restricted by a particular metabolic–epigenetic architecture [7,8].

### 3.4. Why Constraint Is Unstable Rather than Permanent

The instability of this constrained state is increasingly supported by recent longitudinal and molecular profiling studies. In a longitudinal Nature Cancer analysis of *IDH*-mutant glioma progression, low-grade tumors were enriched for slow-cycling oligodendrocyte progenitor cell-like (OPC-like) malignant states, whereas progression was accompanied by expansion of more proliferative neural progenitor cell-like (NPC-like) populations [7]. Importantly, this transition coincided with a shift from methylation-centered regulatory programs toward increasingly dominant genetic drivers. These findings imply that malignant transformation is not just the addition of “more mutations” to the same tumor blueprint. Rather, transformation may reflect a point at which the original epigenetic framework becomes progressively unable to restrict proliferative and developmentally plastic programs. Complementary work in *IDH*-mutant astrocytoma has likewise linked malignancy to large-scale epigenetic reorganization and reactivation of embryonic development genes, reinforcing the view that aggressive progression is tied to breakdown of an earlier, more constrained cellular identity [7,9,10].

## 4. Selective Pressures That Destabilize the Constrained State

### 4.1. Therapy as an Evolutionary Force

If epigenetic constraint helps to define the early biological state of many LGGs, the next question is what destabilizes that state. Malignant transformation does not occur in a vacuum. It emerges under the cumulative influence of selective pressures that favor subclones or cell states with higher adaptability, proliferative fitness, or stress tolerance. Among these pressures, therapy is particularly important. Temozolomide (TMZ), although widely used and clinically valuable, also acts as an evolutionary force. Temozolomide may influence progression not only through cytotoxicity but also through treatment-associated mutagenesis. In subsets of *IDH*-mutant glioma, prolonged alkylator exposure has been linked to mismatch repair-deficient hypermutation, increased mutational burden, and recurrence patterns associated with high-grade transformation and poorer post-transformation survival. In this context, the mutagenic consequences of TMZ refer to its potential to expand the genetic diversity of surviving tumor cells and thereby increase the pool of variants available for subsequent selection [12,13].

### 4.2. Hypoxia and Vascular Stress

Hypoxia and vascular stress represent a second major class of selective pressure. Even before formal histologic transformation, LGGs are exposed to spatially heterogeneous oxygenation, nutrient limitation, and vascular adaptation. These stresses can select for cells capable of metabolic flexibility, stress-response activation, and ecological niche exploitation. Although most mechanistic work on vascular plasticity has been performed in glioblastoma, these studies should be regarded primarily as sources of indirect mechanistic insight rather than direct evidence for malignant transformation in LGGs. The vascular microenvironment is not merely a source of nutrients; it is an active signaling compartment that shapes stemness, invasion, endothelial mimicry, immune interactions, and therapy response. As tumors outgrow pre-existing support structures or encounter fluctuating hypoxic stress, those clones that can couple angiogenic signaling, perivascular niche dependence, and invasive behavior gain a selective advantage [14]. Thus, vascular adaptation should not be treated as a late morphologic byproduct of progression. It is better regarded as one of the ecological forces that progressively selects for aggressive phenotypes [14].

Mechanistically, hypoxia-related adaptation in glioma is commonly linked to hypoxia-inducible factor (*HIF*)-dependent transcriptional programs and downstream pro-angiogenic mediators such as vascular endothelial growth factor (*VEGF*) [14,15,16]. These pathways can promote angiogenesis, alter metabolic adaptation, and support the survival of tumor cells under oxygen- and nutrient-limited conditions. In lower-grade glioma progression, direct evidence remains more limited than in glioblastoma; however, these signaling programs provide a plausible mechanistic basis through which hypoxia and vascular stress may contribute to state transition, niche remodeling, and selection of more aggressive phenotypes. In this setting, factors such as *VEGF* and *ANGPT2* may be viewed as candidate mediators linking ecological stress to vascular adaptation and malignant evolution [14,17].

### 4.3. Immune Remodeling and Immune Editing

Immune remodeling also imposes selective pressure on evolving gliomas. The immune microenvironment in glioma is neither static nor uniformly suppressive from the earliest stages. Instead, available evidence suggests a progressive co-evolution in which the cellular composition and functional state of immune infiltrates shift alongside tumor-intrinsic state changes. Myeloid dominance, T-cell exclusion or dysfunction, altered cytokine signaling, and changes in antigen presentation may all contribute to the selective survival of tumor populations better equipped for immune evasion. A recent review in *Journal for ImmunoTherapy of Cancer* emphasized that glioma cellular states and immune states evolve together and that these changes can vary with tumor stage, intrinsic phenotype, and recurrence setting [18]. In the context of LGG transformation, this means that immune pressure is not simply an external barrier to tumor growth; it is one of the evolutionary filters through which more aggressive tumor states are selected and stabilized [18].

### 4.4. Genomic and Epigenomic Instability

A fourth destabilizing force is genomic and epigenomic instability itself. A recent review in *Acta Neuropathologica* highlighted how chromosomal instability, mismatch repair defects, microsatellite instability, and broader epigenetic instability contribute to progression and aggressive behavior in IDH-mutant astrocytoma [10]. This matters because instability expands the range of available phenotypic solutions. As heterogeneity increases, the tumor is more likely to generate cell populations capable of stress adaptation, treatment resistance, stemness maintenance, or enhanced invasion. From this perspective, malignant transformation is not a pre-scripted endpoint encoded at diagnosis; it is a dynamic process in which persistent stress, incomplete killing, and accumulating instability interact to enlarge the adaptive landscape available to the tumor [10,12,13].

Taken together, these selective pressures should not be viewed as isolated or strictly parallel processes. Rather, they interact across time and biological scale [6,10]. Therapy may impose a selective bottleneck while simultaneously increasing mutational diversification; hypoxia and vascular stress may favor stress-adapted and invasive states while also reshaping immune and metabolic conditions; immune remodeling may further stabilize aggressive states that have already gained a fitness advantage under therapeutic or ecological stress; and genomic or epigenomic instability may broaden the repertoire of tumor states available for subsequent selection. In this sense, malignant transformation is unlikely to result from any single pressure acting alone. Instead, it more plausibly reflects iterative feedback between tumor-intrinsic adaptation and ecosystem-level remodeling, with sequential and synergistic interactions progressively destabilizing the constrained state [6,10].

## 5. Cell-State Transitions as the Proximate Engine of Transformation

### 5.1. From Lineage Restriction to Developmental Plasticity

Although selective pressures create the conditions for malignant evolution, they do not by themselves explain how aggressive phenotypes are executed. The most immediate mechanistic substrate of transformation appears to be cell-state transition. This distinction is important. Genetic alterations define what a tumor may be capable of, but cellular states define what it is currently doing under a given set of internal and external conditions. In progressing *IDH*-mutant gliomas, recent single-cell and chromatin-resolved studies have shown that progression in *IDH*-mutant gliomas is associated with shifts away from relatively differentiated glial-like states toward more proliferative, stem-like, and developmentally plastic phenotypes. These observations are most strongly supported by recent integrated profiling of *IDH*-mutant glioma progression, and are further consistent with treatment-response studies showing depletion of stem/progenitor-like populations during lineage differentiation induced by mutant *IDH* inhibition [7,19,20,21]. These state changes are not incidental. They are the biological form through which selection is translated into increased malignancy.

One major dimension of this process is altered lineage identity. The *Nature Cancer* study of progressing *IDH*-mutant gliomas showed expansion of NPC-like malignant cells as tumors advanced, along with evidence that low-grade lesions were relatively enriched for more slowly cycling OPC-like populations [7]. This matters because lineage state is closely tied to proliferation, transcriptional wiring, and niche interaction. A tumor dominated by slowly cycling, lineage-constrained states may still be clinically serious, but it occupies a narrower adaptive regime than one enriched for progenitor-like or stem-like cells capable of responding flexibly to hypoxia, therapy, and microenvironmental stress. Malignant transformation, in this sense, may be viewed as a lineage problem as much as a mutational one: aggressive disease emerges when tumors become less constrained by mature glial identity and more permissive of progenitor-like, plastic, or stemness-associated programs [7].

### 5.2. Acquisition of Stem-like and Stress-Adaptive Phenotypes

A second dimension is the acquisition or expansion of stem-like features. The language of “cancer stem cells” can sometimes oversimplify heterogeneous malignant ecosystems, but the core idea remains relevant: progression is strongly associated with enrichment of self-renewing, therapy-tolerant, and developmentally plastic cell populations. In the GLASS study, hypermutation and acquired *CDKN2A* loss were linked to increased proliferative neoplastic states at recurrence [6]. The related literature has also underscored that recurrent gliomas often show enrichment of stemness-associated transcriptional programs, reduced differentiation signatures, and greater tolerance to cellular stress. These changes are biologically plausible drivers of malignant transformation because they simultaneously support repopulation, invasion, and therapeutic persistence. They also provide a bridge between the older stem-cell hypothesis and the newer cell-state framework: rather than viewing stemness as a fixed subpopulation trait, transformation may involve broader state shifts that increase the fraction of tumor cells with stem-like functional behavior [6,7].

A third dimension is stress adaptation. As tumors evolve under hypoxia, alkylator exposure, oxidative stress, and immune pressure, those cells able to upregulate survival programs and maintain fitness under adversity are preferentially retained. Recent work in *IDH*-mutant astrocytoma has shown that increasing malignancy is associated with epigenetic landscape reorganization and reactivation of embryonic developmental genes, alongside transcriptional programs related to cell cycle, extracellular matrix interaction, and reduced glial differentiation [9]. These observations suggest that transformation is not only a matter of “more proliferation”; it involves a larger-scale rewiring of developmental and stress-response circuits. Cells that adapt successfully to a deteriorating ecological context become the substrate from which aggressive recurrence emerges [9,10].

### 5.3. Why Cell-State Transition Is More Informative than Grade Alone

This cell-state perspective also helps to explain why malignant transformation often precedes its own formal diagnosis. Histopathologic grade is an endpoint classification, whereas state transitions begin much earlier. A tumor may already be undergoing lineage drift, dedifferentiation, niche reprogramming, and therapy-driven selection long before it satisfies criteria for higher-grade disease. This mismatch between biology and morphology is one reason why transformation can appear abrupt clinically even though its underlying molecular evolution has been gradual. Framing transformation around cell-state transitions therefore offers a more temporally realistic model of disease progression than conventional grade-based narratives alone [1,7,10].

### 5.4. Methodological Limitations and Alternative Interpretations

Although recent longitudinal, single-cell, and spatial studies have substantially advanced the understanding of malignant progression in *IDH*-mutant gliomas, these data should not be interpreted as fully definitive. Single-cell analyses are subject to several important limitations, including sampling bias, underrepresentation of spatially diffuse or treatment-altered regions, uncertainty in state annotation, and dependence on computational frameworks used to define cell identities and trajectories [22,23]. As a result, apparent state transitions may, in some cases, reflect shifts in the relative abundance of pre-existing populations rather than true lineage conversion or plasticity at the single-cell level [23].

Longitudinal studies also face inherent constraints. Matched primary–recurrent samples are often limited in number, may reflect treatment-selected populations, and may not fully capture the temporal continuity of progression between sampling points. In addition, histologic progression, molecular evolution, and ecological remodeling do not necessarily occur at the same pace, making causal inference difficult. More broadly, alternative interpretations remain possible, including models in which malignant transformation is driven primarily by selective expansion of pre-existing aggressive clones rather than by broad state plasticity. For these reasons, the framework proposed here should be regarded as a working model that integrates current evidence, while remaining open to revision as more direct longitudinal and functional data become available.

## 6. Microenvironmental Evolution and Ecosystem Rewiring

### 6.1. Vascular Remodeling and the Perivascular Niche

Tumor evolution in LGGs is inseparable from microenvironmental evolution. A transformation framework that focuses only on tumor-intrinsic changes risks underestimating the extent to which malignant progression depends on ecological rewiring. The glioma microenvironment includes vascular cells, immune populations, extracellular matrix, neural circuitry, and region-specific stromal conditions, all of which change over time and influence selective fitness. Rather than viewing the microenvironment as a passive backdrop, it is more accurate to regard it as a dynamic ecosystem that both constrains and amplifies tumor evolution. The term ecosystem rewiring is particularly useful here, because it emphasizes that progression involves reciprocal rather than one-directional change [6,14,18].

The vascular compartment is especially important. Most vascular biology in diffuse glioma has been developed in glioblastoma, where angiogenesis, vascular co-option, endothelial remodeling, perivascular niche signaling, and vascular mimicry are well recognized. Because IDH-mutant lower-grade gliomas are biologically distinct from glioblastoma, these vascular mechanisms should not be assumed to operate identically across disease contexts. In the present Review, glioblastoma-derived studies are cited to illustrate candidate ecological and vascular principles that may inform LGG progression, while recognizing that direct validation in lower-grade gliomas remains limited. Even so, these ideas remain highly relevant to LGG transformation. As tumors progress, vascular remodeling can contribute to regional hypoxia, altered permeability, metabolic gradients, immune trafficking changes, and direct support of stem-like tumor states. The perivascular niche, in particular, has emerged as a microenvironmental compartment that supports tumor cell survival, plasticity, and resistance. Once vascular remodeling becomes coupled to malignant cell-state change, progression may become self-reinforcing: hypoxia selects for aggressive states, aggressive states reshape the vasculature, and the remodeled vasculature then stabilizes those same states [14].

### 6.2. Co-Evolution of Tumor Cells and the Immune Microenvironment

The immune compartment evolves in parallel. Glioma-associated microglia and macrophages, lymphoid exclusion, cytokine signaling gradients, and local antigen presentation all change over time. The review by Elguindy and colleagues argues that immune cell composition and function are continually reshaped by tumor stage and intrinsic glioma states, while glioma phenotypes simultaneously adapt to those immune changes [18]. This reciprocal logic is particularly relevant to malignant transformation. Immune pressure may not simply eliminate susceptible tumor cells; it may preferentially spare those with enhanced immunomodulatory capacity, altered interferon signaling, or stronger ability to create local immune privilege. Once established, these changes can synergize with vascular remodeling and extracellular matrix reorganization to favor invasion and recurrence [18].

### 6.3. Extracellular Matrix Reorganization

The extracellular matrix (ECM) is another major but underappreciated component of transformation biology. Glioma progression is accompanied by active remodeling of a self-advantageous ECM that influences migration, survival signaling, intercellular communication, and therapeutic response. A recent *BMC Cancer* review emphasized that gliomas do not merely exist within a preformed stromal architecture; they actively restructure it to promote recurrence and progression [24]. In the context of LGG transformation, ECM remodeling may contribute to malignant progression in at least three ways: by altering tissue mechanics and invasion routes, by shaping vascular and immune behavior, and by reinforcing dedifferentiated or stem-like tumor states through integrin-mediated and matrix-associated signaling. Importantly, recent transcriptional work in *IDH*-mutant astrocytoma has linked malignancy-associated epigenetic reorganization to increased ECM-related programs, suggesting that matrix remodeling is not just a secondary structural change but part of the transcriptional architecture of transformation [9,24].

### 6.4. Neural Interactions and Integrated Ecosystem Dynamics

Neural interactions may also become more important as transformation proceeds. In the GLASS study, recurrent *IDH*-wild-type gliomas showed increased neuronal signaling programs, raising the possibility that neuron–tumor interactions can contribute to progression-associated invasiveness [6]. Although this area is better developed in glioblastoma than in LGGs, the broader implication is that the brain is not a neutral organ background. Activity-dependent signaling, synaptic-like communication, and region-specific neural environments may shape which tumor states are selected during recurrence and transformation. As the field moves toward more integrated models of glioma ecology, it is likely that vascular, immune, ECM, and neural interactions will increasingly be understood as intersecting components of the same evolving ecosystem rather than separate mechanistic domains [6,14,15,16,17,18,22]. The major biological layers discussed above and their translational implications are summarized in Table 1.

## 7. Malignant Transformation as a Continuum Rather than a Discrete Event

### 7.1. Why Transformation Is Not a Discrete Event

A major implication of the preceding sections is that malignant transformation should not be conceptualized as a binary event. Traditional language often implies that a lower-grade glioma is stable until the moment it “becomes” high grade. However, longitudinal molecular studies argue strongly against such a stepwise view. Instead, transformation appears to unfold as a continuum marked by progressive cell-state redistribution, selective enrichment of adaptive clones, and ecosystem remodeling. Histologic progression remains clinically indispensable, but biologically it may capture only a relatively late stage in a much longer process [6]. This view is further supported by histologically defined recurrent IDH-mutant glioma cohorts, in which malignant transformation was observed in a substantial proportion of cases and was associated with inferior post-transformation outcomes [28,29].

This continuum model helps to explain why transformation risk is so difficult to predict using any single biomarker. Tumor size, contrast enhancement, growth velocity, methylation subtype, proliferation index, treatment exposure, and microenvironmental features may each carry information, but none fully captures the dynamic interplay among state restriction, selection, and escape. If transformation is indeed the cumulative consequence of epigenetic destabilization plus ecological adaptation, then no single “switch” is likely to define it in all tumors. Instead, transformation is better understood as an evolving probability state shaped by multiple biological layers, with important implications for longitudinal monitoring: longitudinal imaging, repeat tissue sampling when possible, methylation drift, treatment-associated mutational signatures, spatially resolved ecosystem change, and emerging liquid biopsy approaches [1,12,13].

### 7.2. Implications for Longitudinal Monitoring

**Figure 2:**
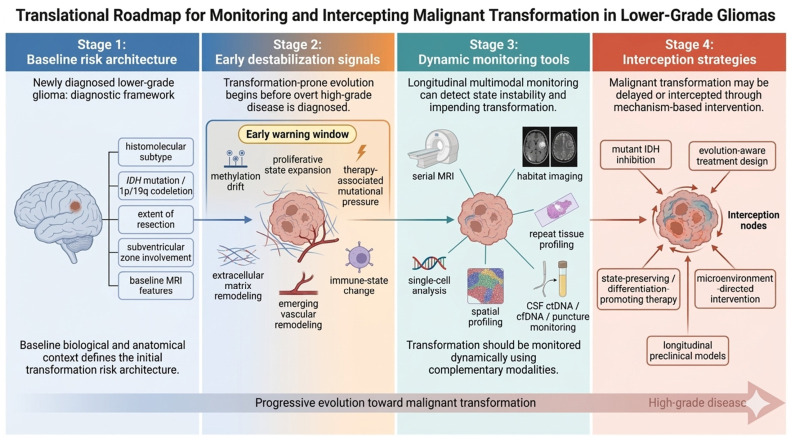
Translational roadmap for monitoring and intercepting malignant transformation in lower-grade gliomas. A transformation-centered framework implies that risk assessment should extend beyond static baseline variables and incorporate dynamic indicators of state instability. At diagnosis, baseline risk architecture is defined by histomolecular subtype, *IDH* mutation/*1p/19q* codeletion status, extent of resection, anatomic context, and MRI features (Stage 1). During disease evolution, early destabilization may be reflected by methylation drift, expansion of proliferative cell states, treatment-associated mutational pressure, and emerging vascular, extracellular matrix, and immune remodeling, thereby creating an early warning window before overt high-grade progression becomes apparent (Stage 2). These changes may be tracked through longitudinal multimodal monitoring, including serial MRI, habitat imaging, repeat tissue profiling, single-cell and spatial analyses, and cerebrospinal fluid ctDNA/cfDNA assessment (Stage 3). Such approaches may support earlier detection of transformation-prone trajectories and guide mechanism-based intervention, including mutant IDH inhibition, evolution-aware treatment design, state-preserving or differentiation-promoting therapy, microenvironment-directed strategies, and longitudinal preclinical models aimed at delaying or intercepting malignant transformation (Stage 4).

This has practical consequences for biomarker development. The most useful transformation biomarkers may not be those that identify a single driver lesion, but those that detect state instability. For example, the expansion of proliferative progenitor-like cell states, emergence of therapy-associated hypermutation, loss of differentiation-associated epigenetic structure, increasing ECM or vascular signatures, and coordinated immune remodeling may collectively provide earlier and more mechanistically grounded warning signals than static baseline markers alone. In this context, the future of malignant transformation monitoring may depend less on one-time risk stratification and more on dynamic surveillance of evolutionary drift [1,9,10]. Recent MRI-based habitat imaging studies further suggest that spatially resolved radiographic ecology can identify high-risk lower-grade glioma subtypes with transformation potential and may support earlier risk stratification [25]. Likewise, longitudinal cerebrospinal fluid cell-free DNA analysis may provide a minimally invasive approach for tracking molecular evolution during treatment and recurrence, thereby complementing imaging-based surveillance [26]. A translational roadmap for monitoring and intercepting transformation-prone evolution is summarized in Figure 2.

## 8. Therapeutic Implications: Preventing State Escape

### 8.1. Current Treatment Modalities and Their Limitations

Current management of lower-grade gliomas is based on a combination of maximal safe resection, radiotherapy, and selected chemotherapy, with treatment decisions increasingly informed by histomolecular classification. More recently, mutant IDH inhibitors have introduced a new mechanism-based option for selected patients with IDH-mutant disease. However, none of these approaches fully eliminates the risk of recurrence or malignant transformation. Surgery is limited by the infiltrative nature of diffuse gliomas, radiotherapy and alkylating chemotherapy may impose long-term toxicity and treatment-associated selective pressure, and even targeted therapies are unlikely to prevent progression in all biological contexts. These limitations underscore the need for a more mechanistic understanding of how epigenetic dysregulation, cell-state plasticity, and ecosystem remodeling shape therapeutic response and transformation risk over time [5,30,31].

### 8.2. State-Preserving Therapy and Mutant IDH Inhibition

A transformation-centered framework also changes how therapy is conceptualized. If malignant progression arises from the erosion of epigenetic constraint and the selection of plastic, adaptive states, then treatment should not be designed solely to shrink tumor burden in the short term. It should also aim to delay or prevent state escape. The recent clinical and regulatory trajectory of mutant *IDH* inhibition illustrates this principle well. In the phase 3 INDIGO trial, vorasidenib improved progression-free survival and delayed time to next intervention in patients with grade 2 *IDH*-mutant glioma [27]. The FDA subsequently approved vorasidenib in 2024 for grade 2 astrocytoma or oligodendroglioma with susceptible *IDH1* or *IDH2* mutations following surgery, marking the first FDA approval of a systemic therapy specifically for this setting [32]. These developments are important not only as therapeutic milestones, but also conceptually: they support the idea that preserving a less aggressive biological state may be a clinically meaningful strategy [27,32].

Mechanistic work further strengthens this interpretation. A recent *Cancer Cell* study showed that mutant *IDH* inhibitors can induce lineage differentiation in *IDH*-mutant oligodendroglioma, deplete stem/progenitor-like populations, and reduce proliferation in responsive tumors [19]. In other words, *IDH* inhibition may work not merely by blocking a metabolic enzyme but by shifting tumor cells back toward more differentiated and less evolutionarily permissive states. This is highly relevant to the present framework. If malignant transformation reflects escape from a constrained state, then therapies that reinforce differentiation, reduce cellular plasticity, or narrow the available adaptive landscape may delay progression even if they do not eradicate every malignant cell [19,27].

### 8.3. Therapeutic Strategies to Delay or Prevent Evolutionary Escape

The same logic should inform how the field thinks about alkylator therapy. TMZ remains central in the management of diffuse glioma, but the accumulating literature on hypermutation and SBS11 makes it increasingly difficult to treat it as a biologically neutral intervention. In some contexts, TMZ may suppress tumor burden while simultaneously creating conditions that favor later evolutionary escape [12,13]. This does not imply that TMZ should be abandoned; rather, it underscores the need for more evolution-aware treatment design. Future approaches may need to integrate molecular subtype, mismatch repair status, treatment timing, and longitudinal monitoring of mutational consequences to identify which tumors are most likely to benefit and which are most likely to be evolutionarily accelerated by exposure [12,13].

Microenvironment-directed therapies also deserve renewed attention in transformation biology. Anti-angiogenic therapy has been disappointing when framed too narrowly as a means of starving tumors. However, if vascular remodeling is understood as part of a broader program of ecosystem stabilization, then interventions aimed at the perivascular niche, hypoxia adaptation, immune trafficking, or matrix remodeling may deserve reconsideration in lower-grade progression settings. Likewise, immunotherapy strategies in glioma have historically faced major challenges, but the transformation framework suggests that patient selection may need to focus more explicitly on evolutionary and ecological state rather than on generic disease category. The relevant question is not simply whether a tumor is “a glioma,” but whether it has entered a specific state of immune exclusion, myeloid dominance, vascular adaptation, or therapy-driven diversification that could be therapeutically exploited [14,18,24].

### 8.4. Preclinical Models for Studying Transformation

Finally, model systems must evolve accordingly. Traditional before-versus-after comparisons cannot fully capture transformation as an ongoing process. The emergence of patient-derived models specifically designed to study malignant transformation in *IDH*-mutant glioma is therefore especially important. A recent *Acta Neuropathologica Communications* study described a patient-derived cell model for malignant transformation in IDH-mutant glioma, offering a platform to analyze progressive change rather than only terminal recurrence [33]. Such systems, especially when integrated with longitudinal patient sampling, single-cell analysis, and spatial profiling, may provide the experimental basis for testing how therapy, microenvironment, and cell-state plasticity interact over time. In a field increasingly defined by evolutionary thinking, the most informative models will be those that reproduce temporal transition rather than static endpoint phenotypes [33].

## 9. Conclusions and Future Perspectives

Current evidence supports a shift away from static, grade-based interpretations of LGG progression. Rather than a simple histopathologic escalation driven by the linear accumulation of genetic alterations, malignant transformation is more plausibly understood as the progressive erosion of an initially constrained tumor state under persistent selective pressure. In this framework, mutant *IDH*-associated metabolic and epigenetic programs help to establish a comparatively restricted early state, whereas therapy, hypoxia, immune remodeling, vascular adaptation, extracellular matrix reorganization, and genomic or epigenomic instability progressively destabilize that state over time. Clinically overt high-grade disease emerges when these pressures are translated into cell-state transitions that favor proliferation, developmental plasticity, invasion, and therapeutic resistance. Taken together, these observations support a model in which malignant transformation represents not a discrete event, but the cumulative outcome of evolutionary selection acting on a shifting tumor-state landscape [8,9,10]. Accordingly, the framework proposed here is intended to organize emerging evidence rather than to imply that a single, universally validated model of LGG malignant transformation has already been established.

This framework has important translational implications. It suggests that the most informative biomarkers of impending transformation may be dynamic indicators of state instability rather than static baseline features alone, and that effective intervention may depend not only on tumor cytoreduction but also on limiting cellular plasticity and treatment-driven evolutionary diversification. In parallel, future progress will likely depend on longitudinal, single-cell, spatial, and liquid-biopsy-enabled approaches capable of capturing transformation as a continuous biological process rather than a late diagnostic endpoint. Viewed in this way, malignant transformation becomes not merely a late complication of LGGs, but a potentially interceptable evolutionary trajectory. By reframing progression as evolutionary escape from epigenetic constraint, this model may provide a more coherent basis for integrating molecular pathology, tumor ecology, and translational neuro-oncology, while helping to identify earlier opportunities for precision monitoring and intervention [19,27,32,33].

## Figures and Tables

**Table 1:** Key biological layers, representative evidence, and translational implications during malignant transformation in lower-grade gliomas.

Biological Layer	Main Characteristics	Representative Features/Examples	Translational Relevance
Epigenetic constraint [7,8]	Early *IDH*-mutant LGGs remain in relatively restricted lineage and proliferative states	*IDH* mutation, D-2HG accumulation, methylation-centered regulation	Explains prolonged indolent phase despite retained malignant potential
Selective pressures [6,10,12,13]	Persistent stresses progressively destabilize the constrained state	Therapy, hypoxia/vascular stress, immune remodeling, genomic instability	Supports evolution-aware risk assessment and treatment design
Cell-state transitions [7,19]	Tumor cells shift toward proliferative, plastic, stem-like, and stress-adapted states	Lineage drift, dedifferentiation, progenitor-like expansion	Provides proximal markers of malignant evolution beyond histologic grade
Ecosystem rewiring [14,18,24]	Tumor progression is reinforced by reciprocal microenvironmental remodeling	Vascular remodeling, ECM reorganization, immune co-evolution	Supports combined tumor-intrinsic and microenvironment-directed intervention
Dynamic monitoring [25,26]	Transformation is better captured as a continuous process than a late event	Serial MRI, habitat imaging, repeat profiling, CSF ctDNA/cfDNA	Enables earlier detection of transformation-prone trajectories
Interception strategies [19,27]	Delaying transformation may require limiting state escape and evolutionary diversification	Mutant IDH inhibition, evolution-aware therapy, longitudinal models	Supports mechanism-based intervention before overt high-grade progression

## Data Availability

No new data were created or analyzed in this study.
